# Inhibition of P38 MAPK Downregulates the Expression of IL-1*β* to Protect Lung from Acute Injury in Intestinal Ischemia Reperfusion Rats

**DOI:** 10.1155/2016/9348037

**Published:** 2016-02-11

**Authors:** De-Yi Zheng, Min Zhou, Jiao Jin, Mu He, Yi Wang, Jiao Du, Xiang-Yang Xiao, Ping-Yang Li, Ai-Zhu Ye, Jia Liu, Ting-Hua Wang

**Affiliations:** ^1^Department of Burn and Plastic Surgery, Guizhou Provincial People's Hospital, Guiyang 550002, China; ^2^Institute of Neuroscience, Kunming Medical University, Kunming 650031, China; ^3^Institute of Neurological Disease, Translational Neuroscience Center, West China Hospital, Sichuan University, Chengdu 610041, China; ^4^Department of Pediatrics, Affiliated Hospital of Guiyang Medical College, Guiyang 550004, China; ^5^Experimental Animal Center, Kunming Medical University, Kunming 650031, China

## Abstract

Acute lung injury (ALI) induced by intestinal ischemia/reperfusion (II/R) has high incidence and mortality, in which IL-1*β* was essential for the full development of ALI. However, the detailed regulating mechanism for this phenomenon remains to be unclear. The purpose of this study was to investigate whether inhibition of P38 MAPK could downregulate the expression of IL-1*β* to protect lung from acute injury in II/R rats. Here, we found that the level of pulmonary edema at 16 hours after operation (hpo) was obviously enhanced compared to that in 8hpo and sham groups. Immunofluorescent staining demonstrated that IL-1*β* and P38 MAPK were detected in lung tissues. And rats with II/R have the highest translation level for IL-1*β* and phosphorylation of P38 MAPK in lung tissues at 16hpo compared with 8hpo and sham groups. Moreover, administration of SB239063, an inhibitor of P38 *α* and *β*, could effectively downregulate the expressions of IL-1*β* and protects lung tissues from injury in II/R rats. Our findings indicate that the inhibition of P38 *α* and *β* may downregulate the expression of IL-1*β* to protect lung from acute injury in II/R, which could be used as a potential target for reducing ALI induced by II/R in the future clinical trial.

## 1. Introduction

Intestinal ischemia/reperfusion (II/R) injury was the common pathological process of multiple clinical severe diseases. II/R not only caused the intestinal local damage [1], but also led to the injury of distant organs [2], especially acute lung injury (ALI) [3]. ALI induced by II/R has high morbidity and mortality in clinic [4, 5]. Previously, the study indicated that the excessive elevation of proinflammatory cytokines is a major contributor in the occurrence and development of ALI caused by II/R. However, the regulated mechanism of proinflammatory cytokines involving in interleukin-1 (IL-1*β*) in ALI induced by II/R is waiting to be elucidated.

IL-1*β*, a member of inflammatory factors, could interact with central endogenous opioid neurotransmitter systems and then induce or perpetuate pathological states such as persistent pain syndromes, depression, substance use disorders, and their comorbidity [6]. It has been reported that one of the most biologically active cytokines in the early phases of ALI is IL-1*β*, which is elevated in plasma, and that IL-1*β* is a potent inducer of lung and causes release of a variety of proinflammatory chemokine, such as monocyte chemotactic protein-1, macrophage inflammatory protein-1*α*, IL-6, and IL-8 with subsequent recruitment of inflammatory cells into the air spaces as well as being able to alter endothelial-epithelial barrier permeability and fluid transport leading to edema [7–11]. Although the expression of IL-1*β* has been detected in lung tissues of rats from liver ischemia reperfusion injury [12], and IL-1*β* was found in lung tissues of rats with ALI induced by II/R, also the expression of IL-1*β* in the ischemic jejunum and the lung tissues treated with II/R was increased [1, 13]. Whether IL-1*β* may be considered as a candidate molecule for regulating ALI induced by II/R and underlying regulating mechanism involved in P38 need to be elucidated.

In this study, we investigated the expression of IL-1*β* in injured lung after intestinal ischemia. Then, by administration of SB239063, an inhibitor of P38 *α* and P38 *β* mitogen activated protein kinase (MAPK), we investigated whether IL-1*β* expression could be regulated by P38 MAPK signal, so as to determine role of IL-1*β* in ALI induced by II/R and relative molecular signal regulation in lung tissues subjected to II/R.

## 2. Materials and Methods

### 2.1. Animal and Grouping

#### 2.1.1. Animal Care

All adult male Sprague-Dawley (SD) rats, weighing 200–250 g, were purchased from animal center of Sichuan University. The guidelines of caring laboratory animals have been described as follows. Briefly, all animal care, breeding, and testing procedures were conformed to the principle of guidance suggestion for the care and use of laboratory animals promulgated by National Institutes of Health. Rats were housed in cages with a 12-h light/dark cycle and have access to food and water. What is more, following intestinal ischemia/reperfusion (II/R) experiment, animals were placed in warm condition (36.5–37°C) using a heat lamp in order to keep their body temperature.

#### 2.1.2. Surgery 1

Adult male SD rats were randomly divided into sham group and intestinal ischemia/reperfusion (II/R) groups (*N* = 2) at 8 h and 16 h postoperation, described in Table 1. The operation was performed according to principles of surgical aseptic. Rats were anesthetized using 3.6% chloral hydrate by intraperitoneal injection and then fixed by prone position. After the skin of rat was disinfected using povidone, then abdomen cavity was opened, then the superior mesenteric artery (SMA) and coeliac artery (CA) were clamped for 40 min at the same time, followed by intestinal reperfusion as described by previous reports [14, 15]. The sham group was performed with similar laparotomy but did not clamp SMA and CA.

#### 2.1.3. Surgery 2

Adult male SD rats were randomly divided into two groups: ordinary II/R (injury) group; inhibitor + II/R (inhibitor) group. Inhibitor +II/R (inhibitor) group accepted P38 *α* and P38 *β* inhibitor SB239063 (10 mg/kg) injection at 1 hour after operation. Afterwards, rats were anesthetized as described above and fixed. Other processes were performed as Surgery 1. The injury groups were performed with similar processes but were not injected by P38 *α* and P38 *β* inhibitor.

#### 2.1.4. Tissue Harvest

Animals performed for Surgery 1 were deeply anesthetized at 8 hours of postoperation (hpo) and 16 hpo, respectively. Then, lung tissues were obtained and stored at −80°C for further use. Moreover, animals subjected to Surgery 2 were sacrificed at 16 hpo; then, their lung tissues were harvested and stored at −80°C for further use.

### 2.2. Observation of Lung Injury

#### 2.2.1. Pulmonary Edema

Wet/dry ratio of lung was used for testing the level of pulmonary edema. The operation of median sternotomy was performed after finishing reperfusion. Then, lung lobe was cut from pleural cavity. The right lung was placed at 90°C drying oven for 24 h after it was weighed. The right lung was weighed again after the accomplishment of drying. Wet/dry ratio of lung was calculated by dividing the wet weight by the dry weight.

#### 2.2.2. ALI Scoring

The lung tissues were sectioned in a freezing microtome (Leica CM1990, Germany), and then the sections were fixed for 30 s and washed for 2 s. The sections were stained using hematoxylin at 60°C for 60 s and then washed in running water for 10 s. Then, the sections were incubated in 1% hydrochloric acid alcohol for 3 s. This was followed by a blue staining using promoting blue liquid for 10 s and then washed using running water for 30 s. Afterwards, 0.5% eosin for 60 s was added to the sections and the sections were washed using distilled water for 2 s. 80% ethyl alcohol for 2 s, 95% ethyl alcohol for 2 s, alcohol for 2 s, phenolic xylene for 3 s, xylene (I) for 3 s, and xylene (II) for 3 s were successively added to the sections. In the end, the sections were mounted using neutral balsam. Morphologic examinations were performed using light microscopy, and ALI scoring was evaluated blindly according to Mikawa's method. ALI scores were evaluated by alveolar congestion, hemorrhage, neutrophilic infiltration, and thickness of the alveolar wall and hyaline membrane formation. The severity of lung injury was scored from 0 to 4 (0, minimal damage; 1, mild damage; 2, moderate damage; 3, severe damage; 4, maximal damage). In each animal, 4 separate lung sections were graded to generate the mean score [16, 17].

### 2.3. Immunofluorescent Staining

The lung tissues were harvested and dehydrated by 30% sucrose overnight and then sectioned at 10 *μ*m thickness in a freezing microtome (Leica CM1990, Germany). The sections were washed three times in 0.01 mol/L PBS for five minutes, and 5% goat serum with Triton X-100 was added on the sections for 30 min to block nonspecific binding site. After the sections were incubated at 4°C in a moist chamber over 18 h with anti-IL-1*β* antibody (Rabbit, PA90563Rao1, 1 : 100) and anti-P38 antibody (Rabbit, SC7149, 1 : 200), respectively, they were washed three times in 0.01 mol/L PBS for 5 minutes. Then sections were incubated with fluorescence-labeled secondary antibody Alexa594 (anti-rabbit, Invitrogen, 1 : 200) at 37°C for 1 h in dark place. After the sections were washed three times in 0.01 mol/L PBS for 5 minutes in dark condition, the nucleus was counterstained with DAPI. In the end, sections were observed by the immunofluorescent microscope, and the immunostaining images were obtained using Leica AF6000, to observe the location of IL-*β* and P38 in lung was observed.

### 2.4. Quantitative RT-PCR

Total RNA was isolated from lung tissues after 8 h and 16 h II/R, and lung tissues from sham groups using the Trizol reagent (Invitrogen) produce cDNA, respectively. Then, quantitative RT-PCR (qRT-PCR) analysis was performed to quantify the level of IL-1*β* mRNA. QRT-PCR of cDNA was performed using the forward and reverse primer sequences of IL-1*β*, forward: GAGCTGAAAGCTCTCCACCT; reverse: TTCCATCTTCTTCTTTGGGT. *β*-actin was as internal control, forward: GAAGATCAAGATCATTGCTCCT; reverse: TACTCCTGCTTGCTGATCCA. Then the reaction was performed at 95°C for 2 min and circulated 40 times at 95°C for 20 s, 50°C for 30 s, and 60°C for 40 s. The fluorescence was collected and recorded after finishing 40 cycles. All reaction was performed on real-time fluorescent quantitative PCR (ABI7300). Data were analyzed using a relative critical threshold (Ct) method where the amount of target normalized to the amount of inner control.

### 2.5. Western Blotting

1 mL precooling protein extraction reagent which contained 98% RIPA lysis buffer (Beyotime, Jiangsu, China) and 2% cocktail pill (Roche) was added to lung tissues and lung tissues were homogenized on ice using in situ homogenate machine so that tissue mass was invisible to the eyes. After the mixture was ice-bathed for 30 min and blended every 10 minutes, the mixture was quashed for 5 s every 5 s, total 10 times in Ultrasonic Cell Breaking Machine. The lysate was centrifuged at 12000 g for 15 min at 4°C; then, the supernatant was collected, and the concentration of protein was curtained by BCA protein assay kit (Beyotime Institute). After that, the precipitated proteins (80 *μ*g) were separated on a SDS-PAGE gel at 350 mA for 2 h and transferred to PVDF membranes at 350 mA for 2 h. SDS-PAGE consisted of 10 mL 15% separation gel and 6 mL 5% spacer gel. 10 mL 15% separation gel consisted of 2.3 mL ddH_2_O, 5 mL 30% polyacrylamide, 2.5 mL 1.5 mol/L Tris (PH8.8), 0.1 mL 10% sodium dodecyl sulfate (SDS), 0.1 mL 10% ammonium persulfate (AP), and 0.004 mL TEMED. 6 mL 5% spacer gel consisted of 4.1 ddH_2_O, 1.0 mL 30% polyacrylamide, 0.75 mL 1.5 mol/L Tris (PH6.8), 0.06 mL 10% SDS, 0.06 mL 10% AP, and 0.006 mL TEMED. After the transfer was finished, the PVDF membranes were washed in 1 × TBS, then placed in 5% nonfat dried milk seen as sealed liquid, and slowly swayed for 2 h on horizontal pendulum table. Then the PVDF membranes were incubated with primary anti-IL-1*β* antibody (Rabbit, PA90563Rao1, 1 : 400), anti-P38 antibody (Rabbit, SC7149, 1 : 200), and anti-P-p38 antibody (Rabbit, SC-101759, 1 : 400) overnight at 4°C, respectively. After the PVDF membranes were rapidly washed three times in 1 × TBST for 5 minutes on horizontal pendulum table, the PVDF membranes were incubated with the secondary antibody Abexcel (anti-rabbit, Abcam, 1 : 1000) for 2 h with bobble at room temperature on horizontal pendulum table. Afterwards, the PVDF membranes were washed three times in 1 × TBST for 5 minutes on horizontal pendulum table and scanned in Alpha Innotech (BioRad) with ECL.

### 2.6. Statistical Analysis

The experimental data was in the form of the mean ± standard deviation (SD) and analyzed using SPSS 13.0. Moreover, data were analyzed by one-way analysis of variance (ANOVA). Probabilities less than 5% (*P* < 0.05) have statistical significance.

## 3. Results

### 3.1. The Change of Pulmonary Edema and Morphology

**Figure 1:**
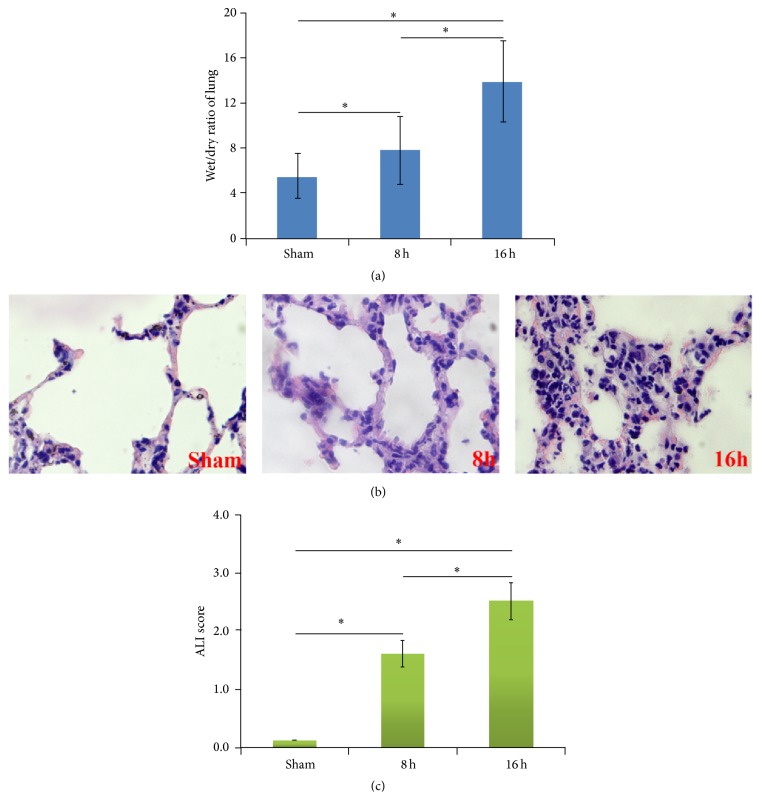
Pulmonary edema and morphology. (a) The results of wet/dry ratio of lung. The data was shown as mean ± SD. ^*∗*^ *P* < 0.05. (b) The results of HE staining. The pictures for sham, 8 hpo, and 16 hpo were shown in left, middle, and right line (original magnification ×400). (c) Results of ALI scores in sham, 8 hpo, and 16 hpo groups.

Wet/dry ratio of lung was measured to detect the degree of pulmonary edema. ALL results showed that rats with II/R or not have a different wet/dry ratio of lung, which revealed a statistical significant uptrend with more treatment time. And rats with II/R have the highest wet/dry ratio of lung at 16 hpo, compared with 8 hpo rats and sham groups, which have significant difference (Figure 1(a)). These data confirmed that the level of pulmonary edema at 16 hpo enhanced greatly than 8 hpo and sham groups. HE staining was used for observing the pulmonary morphology. The results demonstrated that there was normal morphology in sham group, in which the structure of alveoli was clear and intact with the epithelium and microblood vessel distribution. Almost no evidence of lung injury was seen in the sham group. However, following the II/R, severe lung injury was found, in which the presence of extensive interstitial edema, severe alveolar hemorrhage, and extensive inflammatory cell infiltration could be seen (Figure 1(b)). The assessment of ALI scores showed that II/R increased the lung histological score. ALI scores in rats with ALI induced by II/R at 8 hpo and 16 hpo were higher than that in sham group (Figure 1(c)).

### 3.2. Subcellular Localization of IL-1*β* and P38 MAPK in Lung Tissues

**Figure 2:**
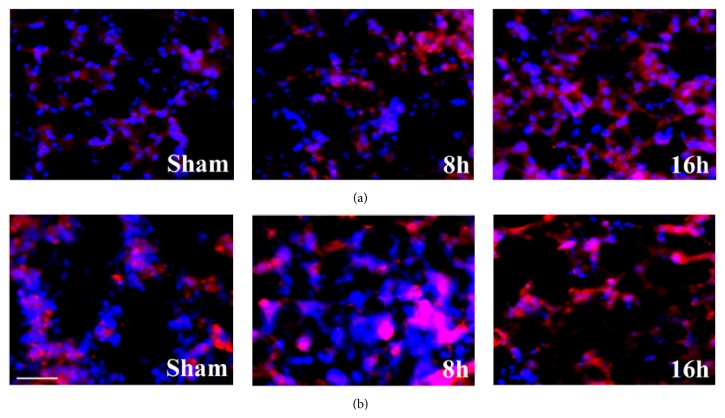
Distribution of IL-1*β* and P38 MAPK in each group. (a) Immunofluorescent staining of IL-1*β*. Il-1*β* was stained red and DAPI was stained blue. (b) Immunofluorescent staining of P38 MAPK. P38 MAPK was stained red and DAPI was stained blue. Scale bar = 50 *μ*m in each picture.

Immunofluorescent staining was performed to detect the distribution of IL-1*β* and P38 MAPK in lung tissues, which showed that, in the lung tissue, the localizations of IL-1*β* and P38 MAPK were mainly found in both epithelia and macrophage (Figures 2(a) and 2(b)).

### 3.3. The Expression Level of IL-1*β* and Phosphorylated P38 MAPK in Lung Tissues

**Figure 3:**
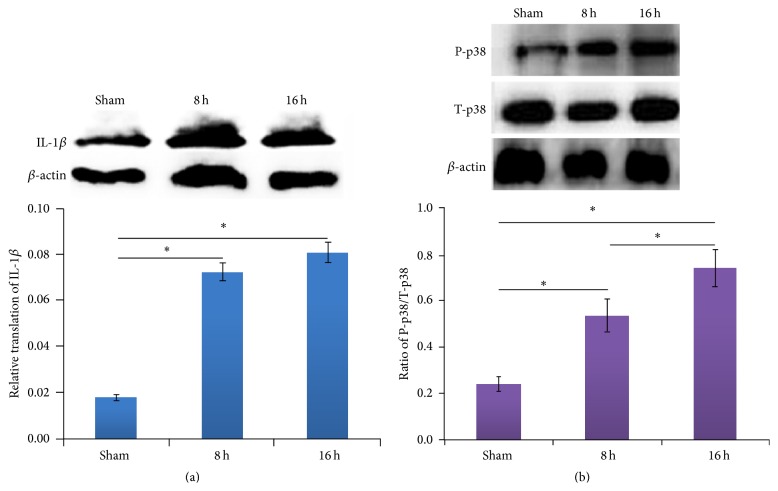
The translation level of IL-1*β* and phosphorylated P38 MAPK in each group. (a) The expression of IL-1*β* protein in experimental groups. ^*∗*^ *P* < 0.05. (b) The expression of phosphorylated P38 MAPK protein in experimental groups. P-p38: phosphorylated p38 MAPK; T-p38: total p38 MAPK. The data was shown as mean ± SD. ^*∗*^ *P* < 0.05.

Western blotting was used for verifying the expression level of IL-1*β* and the level of phosphorylated P38 MAPK in lung tissues. The results showed that rats with II/R are different from sham one on the level of IL-1*β* expression. A statistical significant rising trend could be seen after injury. Rats with II/R have the highest IL-1*β* translation level in lung tissues at 8 hpo and 16 hpo, compared with sham group (Figure 3(a)). Moreover, the phosphorylation of P38 MAPK was the highest at 16 hpo compared with 8 hpo and sham group (Figure 3(b)).

### 3.4. Effect of P38 *α* and P38 *β* Inhibitor for Change of IL-1*β*

**Figure 4:**
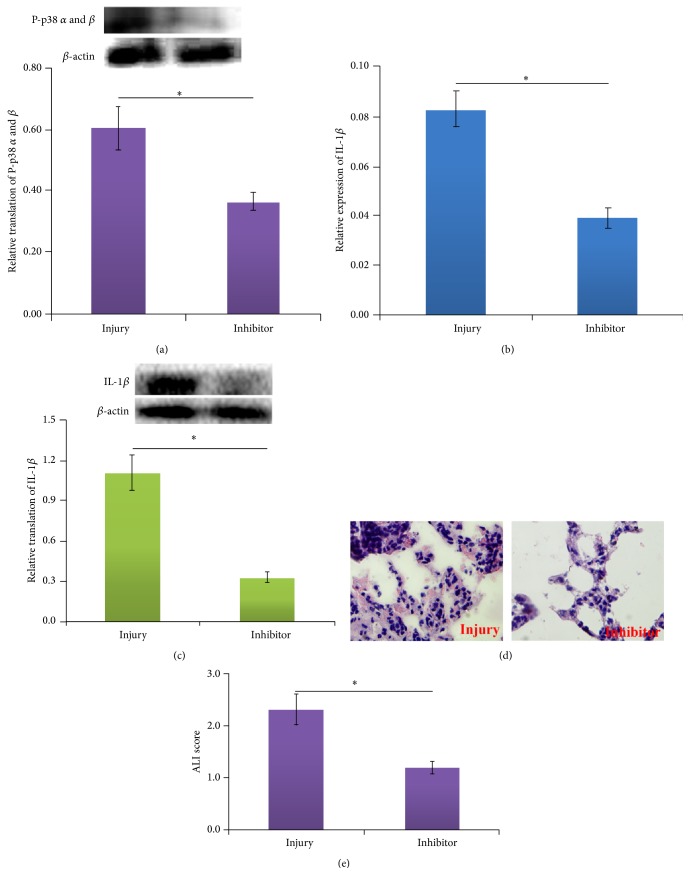
The results of addition of SB239063, an inhibitor of P38 *α* and *β*. (a) The expression of P38 *α* and *β* after addition of SB239063. ^*∗*^ *P* < 0.05. (b) The mRNA expression of IL-1*β* after addition of SB239063. (c) The protein expression of IL-1*β* after addition of SB239063. The data was shown as mean ± SD. ^*∗*^ *P* < 0.05. (d) Pictures of HE staining for injury and inhibitor group (original magnification ×400). (e) ALI scores in sham, injury, and inhibitor group. ^*∗*^ *P* < 0.05.

P38 *α* and P38 *β* inhibitor SB239063, designed and purchased from company, was applied for interfering in the protein expression of P38 *α* and P38 *β*. The results showed that, in rats injected by P38 *α* and P38 *β* inhibitor (inhibitor group), low expression level of P38 *α* and P38 *β* could be detected, when compared with those of no injection one (injury group) (Figure 4(a)). As a result, the mRNA expression for IL-1*β* was correspondingly decreased in rats subjected to P38 *α* and P38 *β* inhibitor administrated rats (inhibitor group), when compared with only II/R groups (injury group) (Figure 4(b)). Moreover, the protein expression of IL-1*β* revealed similar result with mRNA expression of IL-1*β* (Figure 4(c)). The effects of P38 *α* and P38 *β* inhibitor on the histopathological changes of lungs in rats with II/R were shown in Figures 4(d) and 4(e). Morphological study showed the lung tissues of rats were badly damaged in injury group, with severe interstitial edema, severe alveolar hemorrhage, and extensive inflammatory cell infiltration, whereas only mild lung edema, hemorrhage, and inflammatory cell infiltration were observed in inhibitor group (Figure 4(d)). The results of ALI scores showed that after administration of P38 *α* and P38 *β* inhibitor, a drastic reduction in pathological score was shown in inhibitor group compared with that in injury group (Figure 4(e)).

## 4. Discussion

In this study, ALI can be caused by II/R, which was similar as in the previous study [18, 19]. The results of immunofluorescent staining demonstrated that IL-1*β* and P38 MAPK were detected in both epithelia and macrophage in lung tissues after II/R. Moreover, the results of western blotting showed that the raising levels of the expression of IL-1*β* and P38 MAPK in lung tissues after II/R were exhibited. Previously, upregulation of IL-1*β* was detected in lung tissues [2], but the activation of P38 MAPK in lung tissues after II/R was reported by a few. In this study, we confirmed activation of P38 MAPK exhibited similar change tendency with IL-1*β*. Importantly, the level of IL-1*β* in lung tissues after II/R was decreased with the administration of P38 *α* and P38 *β* inhibitor, which indicated that the decrease of the level of IL-1*β* expression may be correlated with inhibition of P38 MAPK signal pathway.

Previous study showed obstruction of thoracic lymphatic flow before II/R decreased the ability of cultured lung tissue explants to release IL-1*β*, IL-10, and VEGF [20], and diminished neutrophil recruitment, as well as reduced concentration of TNF-*α* and IL-1*β* level [21]. There were different changes in the level of protein and gene expression for IL-1*β* after treatment and phospholipase A(2) inhibitor chloroquine or cyclooxidase inhibitor indomethacin after injury, respectively [22]. IL-1*β* increase may be the result of the lung injury induced by II/R, whereas, in this study, significantly, we found P38 inhibitor is useful to downregulate IL-1*β*, which is an important finding to elucidate the mechanism of P38 MAPK signal pathway involving in IL-1*β* regulation. Although it has well been known P38 MAPK plays a crucial role in organ damage after II/R [23–25], no report showed that inhibition of P38 can influence IL-1*β* in rats with ALI after II/R. Our results provide the novel information, in which P38 inhibition to suppress IL-1*β* may be a useful strategy for the treatment of ALI. Previously, most of views thought that IL-1*β* could induce P38 MAPK activation in gastric adenocarcinoma [26], and pretreatment of cells with tangeretin could inhibit IL-1*β*-induced P38 MAPK in human lung carcinoma cells [27]. Moreover, other opinions indicated that P38 MAPK can activate nuclear factor- (NF-) *κ*B to induce IL-1*β* production in rat pulmonary interstitial macrophages [28]. Comparing these findings, we found inhibitor of P38 MAPK decreased effectively the level of IL-1*β* expression and significantly improved the lung injury, which may be important to give a new cure by administration of P38 MAPK to interfere ALI after intestinal ischemia reperfusion. It has been known that IL-1*β* is a bad molecule that is not convenient recovery [29–31]; therefore, decreased IL-1*β* expression should be better for the recovery of lung injury. To our knowledge, this is the first study to show a protective effect of inhibition of P38 *α* and *β* MAPK on ALI induced by II/R. Our results give important implication to explore the mechanism of lung injury in rats subjected to II/R, which is involved in P38 MAPK regulating IL-1*β*. This is different from previous report that IL-1*β* directly regulates P38.

In conclusion, II/R can increase the levels of expression of IL-1*β* and P38 MAPK in ALI, and the level of IL-1*β* expression and the severity of ALI can be decreased by P38 *α* and *β* inhibitor SB239063. This fundamental information may provide a potential strategy to target directly P38 or IL-1*β* for the treatment of reducing ALI induced by II/R in the future clinical trial.

## Figures and Tables

**Table 1:** Animal model and sample harvest.

Groups	*N*	Treatment	Following process
Sham	8	/	Lung tissues were harvested at 8 hours
8 hpo	8	II/R	Lung tissues were harvested at 8 hours after II/R
16 hpo	8	II/R	Lung tissues were harvested at 16 hours after II/R
